# Optimization of Polyphenol Extraction from Purple Corn Pericarp Using Glycerol/Lactic Acid-Based Deep Eutectic Solvent in Combination with Ultrasound-Assisted Extraction

**DOI:** 10.3390/antiox14010009

**Published:** 2024-12-25

**Authors:** Ravinder Kumar, Sherry Flint-Garcia, Miriam Nancy Salazar Vidal, Lakshmikantha Channaiah, Bongkosh Vardhanabhuti, Stephan Sommer, Caixia Wan, Pavel Somavat

**Affiliations:** 1 Food Science Program, Division of Food, Nutrition and Exercise Sciences, University of Missouri, Columbia, MO 65211, USA; rkz8m@missouri.edu (R.K.); lchannaiah@missouri.edu (L.C.); vardhanabhutib@missouri.edu (B.V.); ssommer@missouri.edu (S.S.); 2 Plant Genetics Research Unit, Agricultural Research Service, United States Department of Agriculture, Columbia, MO 65211, USA; sherry.flint-garcia@usda.gov; 3 Division of Plant Sciences and Technology, University of Missouri, Columbia, MO 65211, USA; salazarvidalm@missouri.edu; 4 Department of Chemical and Biomedical Engineering, University of Missouri, Columbia, MO 65211, USA; wanca@missouri.edu

**Keywords:** purple corn pericarp, polyphenols, extraction optimization, response surface methodology, deep eutectic solvents, ultrasound-assisted extraction, antioxidant properties

## Abstract

Purple corn pericarp, a processing waste stream, is an extremely rich source of phytochemicals. Optimal polyphenol extraction parameters were identified using response surface methodology (RSM) by combining a deep eutectic solvent (DES) and ultrasound-assisted extraction (UAE) method. After DES characterization, Plackett–Burman design was used to screen five explanatory variables, namely, time, Temp (temperature), water, Amp (amplitude), and S/L (solid-to-liquid ratio). The total anthocyanin concentration (TAC), total polyphenol concentration (TPC), and condensed tannin (CT) concentration were the response variables. After identifying significant factors, the Box–Behnken design was utilized to identify the optimal extraction parameters. The experimental yields under the optimized conditions of time (10 min), temperature (60 °C), water concentration (42.73%), and amplitude (40%) were 36.31 ± 1.54 g of cyanidin-3-glucoside (C3G), 103.16 ± 6.17 g of gallic acid (GA), and 237.54 ± 9.98 g of epicatechin (EE) per kg of pericarp, with a desirability index of 0.858. The relative standard error among the predicted and experimental yields was <10%, validating the robustness of the model. HPLC analysis identified seven phytochemicals, and significant antioxidant activities were observed through four distinct assays. Metabolomic profiling identified 57 unique phytochemicals. The UAE technique combined with DES can efficiently extract polyphenols from purple corn pericarp in a short time.

## 1. Introduction

In recent times, environmentally friendly solvents and highly efficient phytochemical extraction techniques are eliciting increasing researcher/industrial interest and so is the demand for economically recovered value-added phytochemical compounds from plant material. Among the various novel extraction techniques explored by researchers, ultrasound-assisted extraction (UAE) has gained considerable popularity for enhancing secondary plant metabolite extraction [1,2,3]. In the domain of environmentally friendly solvents, the potential of deep eutectic solvents (DESs) has witnessed extensive explorations [4,5,6]. DESs are considered environmentally friendly solvents and are made up of two molecules, where one is a hydrogen-donating molecule, and the other is a hydrogen-accepting molecule. First identified by Abbott et al. [7], these solvents behave similar to ionic liquids and entail various potential applications [8]. Several tailor-made DES have been reported in the literature for their distinctive applications. Their uses range from the extraction of metabolites from different plant materials, chromatographic separation of natural products, drug delivery systems, and as electrochemical reagents, besides many other novel industrial applications [9]. DESs have been utilized to extract phenolic acids, flavonoids, and anthocyanins from various plant matrices, such as grape skin [5], green tea [6], *Cajaus cajan* [10], strawberry [11], and blueberries [4]. The benefits of DESs over conventional solvent systems have been explored in various studies that report them to be biodegradable, exhibiting improved extraction efficiency in a shortened time, are able to extract compounds of varying polarities [12], experience lesser volatility, and are non-flammable [13]. However, the DESs themselves are not able dissolve all the bioactive compounds out of the complex plant matrices and need external aid to efficiently extract the compounds of interest. Therefore, the DESs are often combined with assisted extraction technologies including UAE, which has been reported to be a highly efficient methodology. UAE has emerged as an efficient extraction technique for bioactive compound recovery in the food industry, facilitating extraction from diverse substrates. Several factors play a role in the sonication process, where the localized pressure and temperature result in the rupture of the cell wall, allowing the solvent to efficiently diffuse inside the generated cavities. This results in reduced extraction times, increased mass transfer, and increased amounts of extraction product [1,2,3,14].

Purple corn or maiz morado is a coloured corn variety that has been documented to contain significant concentrations of phytochemicals, most of which are concentrated in the outermost bran layer of the kernel, also called the pericarp [15]. Coloured corn pericarp, a cellulose- and hemicellulose-rich material is a low-value process stream for the industry, which can be separated from the kernel endosperm and selectively processed for the efficient extraction of various phytochemicals [15,16,17]. Therefore, coloured varieties present an exciting opportunity for value addition when it comes to traditional corn farmers, processors, and the food industry in the US. Midwest-adapted coloured corn varieties and their coproducts may find numerous value-added agro-industrial applications compared to the traditional yellow dent corn whose current uses range from the production of starch, modified sweeteners, and ethanol to different sized grits and corn oil. Purple corn pericarp contains manifold higher polyphenol contents compared to some of the other well-known sources, such as blueberries, strawberries, and grapes [18,19]. Researchers have already demonstrated that the lower value pericarp coproduct from corn processing can serve as a potential source of natural red food colorants [20]. In an another optimized extraction study using traditional solvents, almost 34.9 g of anthocyanins/kg of pericarp was quantified, in addition to the copious amounts of other phytochemicals, such as phenolic acids and tannins [16]. The efficacy of using advanced statistical designs such as the Box–Behnken design (BBD) and Placket–Burman design (PBD) for the evaluation of multiple extraction parameters at the same time has already been demonstrated [16,17]. For screening significant parameters, PBD was chosen as it can evaluate two to forty-seven parameters, requiring significantly lesser number of runs. Since PBD is better suited for screening, further optimization using response surface methodology (RSM) was performed with the BBD with three different levels of factors, i.e., −1, +1, and 0. This design can work with a number of factors ranging from three to twenty-one and is easy to use, as it is an independent quadratic design and does not require any additional factorial design in the study [21].

The overarching aim of this work was to use RSM to optimize phytochemical extraction from purple corn pericarp using a previously identified deep eutectic solvent in conjunction with the ultrasound-assisted extraction technique. Although various researchers have used such optimization approaches to extract bioactive compounds from other biological materials, none of those materials contain the extremely high amounts of anthocyanins, condensed tannins, phenolic acids, and flavonoids present in purple corn pericarp. Since pericarp is a corn processing waste stream, efficient valorisation of coloured corn pericarp for value-added bioactive recovery is a contribution to the circular bioeconomic paradigm. Lastly, this research work is the first instance of the optimization of polyphenol extraction from purple corn pericarp using a combination of a select DES formulation and UAE technique, utilizing a preliminary screen of explanatory variables using PBD followed by extraction optimization using BBD.

## 2. Materials and Methods

### 2.1. Materials

The purple corn sample used in this study originated from South America and was purchased from a commercial vendor in 2023 and stored at −18 °C until processed (Woodland Foods, Waukegan, IL, USA). Pericarp from the corn kernels was recovered following a previously reported lab-scale dry milling method [22]. The separated pericarp was ground using a benchtop grinder (ALD Kitchen Professional Grinder, Model No. A-S400G, Amazon USA, Seattle, WA, USA) and passed through a 500 µm mesh screen, and the sieved product was packed and refrigerated at −4 °C until further analysis. Glycerol (99+%) and lactic acid were procured from ThermoFisher Scientific (Waltham, MA, USA). Folin–Ciocalteu reagent, vanillin, sodium phosphate monobasic, sodium carbonate, iron (III) chloride hexahydrate, DPPH, ABTS^+^, ethanol, methanol, di-sodium hydrogen phosphate, and potassium peroxydisulfate were obtained from Sigma Aldrich (St. Louis, MO, USA). Various standards that were used for the chromatographic analysis using HPLC included cyanidin chloride, cyanidin-3-glucoside, malvidin, delphinidin, and peonidin for anthocyanins, followed by epicatechin, kaempferol, morin, naringin, and quercetin for the flavonoid profile, whereas caffeic acid, chlorogenic acid, ferulic acid, gallic acid, and hesperidin were the standards used for phenolic profiling. The mobile phases used for various analyses and spectrophotometric measurements were bought from Sigma-Aldrich (St. Louis, MO, USA). The DI water used for the experiments was obtained from Milli-Q H_2_O purification system (Millipore^®^, Burlington, MA, USA). All reagents used were of analytical grade and the solvents used for the experiments were not subjected to any processing before usage.

### 2.2. Deep Eutectic Solvent Preparation

The most efficient deep eutectic solvent (DES) combination from a previous study of nineteen distinct DES formulations was selected for the optimization work and consisted of glycerol as the hydrogen bond donor (HBD), and lactic acid as the hydrogen bond acceptor (HBA). Both the compounds were measured accurately in a molar ratio of 2:1 (HBD/HBA) and poured in a covered glass beaker that was placed in an oil bath with a magnetic stirrer set at 80 °C. The mix was stirred for 1 h, after which a transparent solvent mixture was formed. This solvent was stored at 4 °C until further use. Heating and stirring at 80 °C is reported to be the method of choice for formulating DESs to extract bioactive compounds from various plant matrices [23,24].

### 2.3. FTIR Analysis

Infrared spectra of individual constituents of the solvent (glycerol and lactic acid), prepared solvent, and formulated solvent mixed with different concentrations of water were evaluated using an ATR-FTIR spectrometer (Nicolet 380, Thermo Scientific, Waltham, MA, USA) for the confirmation of hydrogen bond formation and to check for any changes in bonds before and after DES formation. The equipment was operated at a 4 cm^−1^ spectral resolution, 400–4000 cm^−1^ wavenumber range, and 32 scans per min [25].

### 2.4. Measurement of Polarity and pH

Nile red was used as a solvatochromic dye for the measurement of the polarity of various solvent mixes. Nile red, utilized as the probe, was prepared as a 5 mM stock solution in 96% ethanol and stored at 4 °C. Subsequently, 20 µL of the stock solution was combined along with 980 µL of the DES, and the peak wavelength was determined within the 400 to 800 nm range using a microplate reader spectrophotometer (Multiskan Sky, Thermo Scientific, Waltham, MA, USA). The polarity was quantified in terms of the molar transition energy (*E_T_* (NR)), as per Equation (1).

(1) $$E_{T}\left(NR\right)=h\times c\times v_{max}\times N_{A}=\frac{28591}{\lambda_{max}}$$

In the given context, h represents Planck’s constant, the speed of light is represented by c, *v_max_* is related to the maximum wavelength absorption, N*_A_* corresponds to the Avogadro constant, and *λ_max_* denotes the dye’s absorption spectrum.

A benchtop pH analyser was used (Thermo Scientific (Waltham, MA, USA) Orion Star A211 pH analyser) after calibration using standard solutions of pH 4.01 and 7.00.

### 2.5. Extraction of Polyphenols

Polyphenolic extraction was carried out with a sonic dismembrator equipped with a 9 mm diameter ultrasound probe (Model 505 Sonic Dismembrator (20 kHz, 500 W), Fisher Scientific, Hampton, NH, USA) immersed in 1/3 depth of the solvent in a 50 mL centrifuge tube held in a temperature-controlled water bath with circulating water. Various extraction parameters, namely, time (min), temperature (°C), water (%, *v*/*v*), amplitude (%), and the solid-to-liquid ratio (S/L), were tested during the preliminary studies to ascertain the ranges of these parameters. Based on previous studies where the pulse duration did not show any significant effect on phytochemical extraction [16,17], a medium range for the pulse duration with a 5 s (on) and 5 s (off) time was chosen. The theory behind the duration of ultrasonic pulsation can be elaborated in two ways. One explanation is that with a greater pulse on time, the sonotrode may get heated, resulting in a rise in the temperature that adversely affects the overall phytochemical extraction. On the other hand, having longer pulse off times may result in a reduced ultrasonic effect on the samples, resulting in a reduced extraction efficiency [26]. The extraction process was followed by the addition of DI water (5 mL) and centrifugation (12,000 rpm, 10 min, 4 °C) using a benchtop centrifuge (Hermle Z-327 K, HERMLE AG, Gosheim, Germany). After centrifugation, vacuum filtration was performed on the extracts using a Whatman^®^ (Maidstone, UK) #4 filter paper, followed by the refrigeration of the supernatant at 4 °C until further analysis.

### 2.6. Experimental Design

#### 2.6.1. Screening for the Significant Variables Using the Plackett–Burman Design (PBD)

Five explanatory variables including time (time), temperature (Temp), water (water), amplitude (Amp), and solid-to-liquid ratio (S/L) were screened with the total anthocyanin content (TAC), total phenolic content (TPC), and total condensed tannin (CT) content extracted being the response variables. These variables and their ranges were selected based on preliminary experiments. The final screening experiment had a total of 13 runs with three varying levels of factors, namely, high (+), medium (0), and low (−). Multivariate regression analysis was performed using the first order polynomial equation as given below

(2) $$Y=\beta_{0}+\sum_{i=1}^{4}\beta_{i}X_{i}$$

Here, *Y* denotes the experiment results; *β_i_* and *β*_0_ stand for the coefficient of linearity and intercept, respectively; and the independent parameters are denoted by *X_i_*.

#### 2.6.2. Optimization Using the Box–Behnken Design (BBD)

After reviewing the screening results, the significant factors identified were, time, water, temperature, and amplitude, which were further optimized with the use of the BBD. Similar to the PBD experiment, three different levels (−1, 0, and +1) were employed for the selected parameters. The responses from the BBD analysis were evaluated using a second-order polynomial equation, as given below

(3) $$Y=\beta_{0}+\sum_{i=1}^{3}\beta_{i}X_{i}+\sum_{i=1}^{3}\beta_{ii}X_{i}^{2}+\sum_{i=1}^{3}\times \sum_{j=i+1}^{3}\beta_{ij}X_{i}X_{j}$$

Here, Y denotes the intercept; furthermore, *β*_0_, *β_i_*, *β_ii_*, and *β_ij_* stand for the intercept, linear, squared coefficient and coefficient of interaction, correspondingly. *X_i_* and *X_j_* were individualistic variables. Other notations were used as follows: the coefficient of determination R^2^, predicted R^2^, lack of fit (LoF), *p*-value, F-value, and adjusted-R^2^.

### 2.7. Quantification of Polyphenolic Compounds

#### 2.7.1. Total Anthocyanin Content (TAC)

Total monomeric anthocyanins were analysed using the pH differential method [27]. The stored extracts (100 µL) were diluted in 100 µL of buffer A prepared using potassium chloride (0.025 M KCl with 1 M HCl for adjusting the pH to 1.0) followed by 100 µL of buffer B prepared using sodium acetate (0.4 M Ch_3_COONa with 1 M HCl for adjusting the pH to 4.5) separately. Later, 200 µL of these solutions was pipetted into a 96-well microplate. Next, a 96-well plate reader (Multiskan Sky, Thermo Scientific, Waltham, MA, USA) was used to measure the absorbance at wavelengths of 520 and 700 nm. The actual values were reported in g of cyanidin-3-glucoside (C3G) equivalents per kg of purple corn pericarp and were calculated using the following equation:

$$TAC=\frac{\left(∆AM_{w}D\times 1000\right)}{\left(0.45\varepsilon_{0}P\right)}$$

Here, ∆*A* stands for the difference between absorbances ([*A*_520nm_–*A*_700nm_] at pH 1 − [*A*_520nm_–*A*_700nm_] at pH 4.5), *M_w_* is the molecular weight (449.2 g/mol), *ε*_0_ denotes the molar extinction coefficient for C3G (26,900 L/mol), *D* stands for the dilution factor, *P* denotes the path length (1 cm), and a factor of 0.45 was used to adapt the method to a 96-well plate reader.

#### 2.7.2. Total Phenolic Content (TPC)

The Folin–Ciocalteu solution method discussed in [20] was used for this analysis. Firstly, gallic acid or the extract (80 µL) was mixed into 400 µL of Folin–Ciocalteu solution (10%), next a 75 mg/mL solution of Na_2_CO_3_ (320 µL) was added. This mixture was kept in the dark for 30 min to react and later transferred to 96-well plates. The samples were analysed using a 96-well plate reader (Multiskan Sky, Thermo Scientific, Waltham, MA, USA) at a wavelength of 765 nm. The results were reported in g of gallic acid (GA) equivalents/kg of pericarp.

#### 2.7.3. Condensed Tannin (CT) Content

For the fresh preparation of 1 mL of vanillin, methanol (3 g/100 mL) was mixed with the extract or catechin standard (200 µL), followed by supplementation with 1 mL of a H_2_SO_4_/methanol solution (30%, *v*/*v*). This mixture was let to react in a water bath at 30 °C for 20 min. Later, the mixture was pipetted (200 µL) into microwell plates and read using a 96-well plate reader at a wavelength of 500 nm. CT contents were reported as g of epicatechin equivalents (EEs)/kg of pericarp [16].

#### 2.7.4. Total Flavonoid Content (TFC)

To quantify total flavonoids, 1 mL of pericarp extract or catechin solution was combined with 4 mL of deionized water, followed by the addition of 300 µL of 5% (*w*/*v*) NaNO_2_. This mixture was allowed to react for 6 min, after which 300 µL of 10% (*w*/*v*) AlCl_3_ was added and allowed to react for an additional 5 min. Next, 4 mL of 1 M NaOH was introduced, and the final volume was adjusted to 10 mL by adding 400 µL of deionized water. After a 15 min incubation period, 200 µL of the mixture was transferred to a 96-well plate for absorbance measurement at 510 nm using a Multiskan Sky plate reader (Thermo Scientific, Waltham, MA, USA). The TFC was expressed as g of catechin equivalents (CEs)/kg of pericarp [28].

### 2.8. Polyphenolic Profiling Using a HPLC

#### 2.8.1. Sample Preparation for HPLC Analysis

The filtered extracts were passed through a 0.22 µm syringe filter and stored in glass vials with PTFE screw caps. The analysis was conducted using an Agilent 1200 series system (Agilent Technologies, Palo Alto, Santa Clara, CA, USA) fitted with a diode array detector (DAD) employing a C18 column (5 µm, 250 mm, 4.5 mm, Avantor^®^, Radnor Township, PA, USA).

#### 2.8.2. Anthocyanin Profile

For anthocyanin profiling, the mobile phases used were 5% formic acid in water and acetonitrile, which were named A and B, respectively. Seven different standards (pelargonidin, malvidin, peonidin, cyanidin chloride, delphinidin, and cyanidin-3-glucoside) were run for the targeted identification of compounds at different retention times. The flow rate was set to 1 mL/min, and the diode array detector was set for detection at a wavelength of 520 nm. A variable gradient was used for the analysis, which began with 5% B for 5 min, followed by 100% B at 40 min, was further maintained at 100% for 5 more min, and returned back to 5% by the end of run at 45 min. The column temperature was kept constant throughout the run at 35 °C and the calculations were reported in terms of mg of anthocyanin compound/g of pericarp [29].

#### 2.8.3. Flavonoid Profile

Flavonoid detection was performed using a method that has been previously reported [30]. The method used a flow rate of 1 mL/min, column temperature of 25 °C, and an injection volume of 20 µL. The two mobile phases used were labelled A (HPLC-grade water with a pH of 2.8) prepared using acetic acid, whereas mobile phase B consisted of 100% acetonitrile. A variable gradient was used for the analysis as follows: 10% B from 0 to 5 min; increased to 23% at 31 min; followed by a 5% increase by 43 min, a further increase to 100% B by 47 min for washing the column, and again switching to 100% A by 50 min to equilibrate the system. The diode array detector was set to detect the compounds at four different wavelengths (261, 280, 320, and 360 nm). The final flavonoid content was ascertained using a standard curve and expressed as mg of the specific compound per g of pericarp.

#### 2.8.4. Phenolic Profile

For phenolic acid detection and quantification, the method described by Kandil et al. [31] was used and consisted of two mobile phases. Mobile phase A was prepared using 0.1% acetic acid mixed with HPLC-grade water, and mobile phase B was prepared using 0.1% acetic acid mixed with pure methanol. The pump was set to a flow rate of 1 mL/min and the column was used at room temperature (25 °C). The flow gradient used was varied, starting with 5% B at 0 min and rising to 20% B by 15 min, further increasing to 40% B by 35 min; followed by 65% and 80% at 42 and 50 min, respectively. Later, the gradient was changed back to 5% B at 52 min and kept the same until 60 min to bring the machine to an equilibrium. The injector was set to inject 10 µL of every sample, and the DAD was set at three variable detection wavelengths (254, 280, and 360 nm). For the calculations, the standards (gallic acid, chlorogenic acid, caffeic acid, ferulic acid, and hesperidin) were run using the same method, and the values are reported as mg of compound/g of pericarp.

### 2.9. Metabolomic Profiling

A comparison of the secondary plant metabolites present in the DES optimized extract and a freeze-dried aqueous extract was performed to evaluate and identify the effects of different solvents/extraction processes on the extracted compounds.

#### 2.9.1. Sample Preparation for Metabolic Profiling

Fresh extracts were prepared for metabolomic profiling. The extraction parameters used were those established during the verification of optimized parameters. The extract was centrifuged at 12,000 rpm for 10 min at 4 °C using a temperature-controlled centrifuge (Hermle Z-327 K, HERMLE AG, Gosheim, Germany), vacuum filtered using a Whatman^®^ #4 filter paper, followed by filtration through a 0.22 µm syringe filter. For comparison, another polyphenolic extract was prepared from the ground pericarp using DI water at a solid-to-liquid ratio of 1:20 by shaking the mix in an incubated shaker set at a temperature of 40 °C and 150 rpm for a time of 24 h. This aqueous extract was centrifuged and vacuum filtered, followed by freeze-drying in a lab-scale freeze dryer (Harvest Right^®^, Salt Lake City, UT, USA). Finally, the dried extract was reformulated in approximately 80% methanol (containing the internal standard umbelliferon) so that the concentration of solutions was 1 mg/mL for the UHPLC-MS analysis.

#### 2.9.2. UHPLC-MS Analysis

An ACQUITY UHPLC system paired with a Bruker Impact II mass spectrometer was utilized to analyse the metabolites present in the DES optimized extract, as well as in the aqueous extract. The system featured a C18 reverse-phase column, used an injection volume of 3 µL, and the column temperature was set at 60 °C. The mobile phases included 0.1% formic acid as phase A and acetonitrile as phase B, with a flow rate of 0.56 mL/min. The wavelengths employed ranged between 190 nm and 600 nm, and were monitored by photodiode array detectors (PDA). The gradient started with 5% mobile phase B at 0 min, progressing to 70% over 30 min, reaching 95% at 33 min, maintaining 95% until 36 min, and returning to 5% at 41 min for re-equilibration. Sodium formate was introduced at the end of each gradient for mass spectrum calibration. Data acquisition was performed in positive ion mode. The data were processed using Bruker’s MetaboScape software Version 5.0, and MS/MS spectra were matched with the exact masses of the identified polyphenols or with a reference library.

The mass spectrometer settings included Funnel 1 and Funnel 2 configured with a RF voltage of 300 volts peak-to-peak (Vpp) each, while the hexapole was set to 150 Vpp. Both the collision energy and ion energy were fixed at 8 electron volts (eV). The transfer time was 100 µs, with a pre-pulse storage time of 5 µs. These configurations were optimized for a mass range of *m*/*z* 100 to 1500. For automated tandem mass spectrometry (MS/MS), three precursor ions were selected with varying collision energies: 15 eV for *m*/*z* 100 to 200, 35 eV for *m*/*z* 200 to 500, and 50 eV for *m*/*z* 500 to 1000. Active exclusion was implemented to eliminate precursors after three spectra, with their subsequent re-inclusion after 0.15 min. The final mass spectrum analysis covered a range from *m*/*z* of 100 to 1500.

### 2.10. Antioxidant Assays

#### 2.10.1. ABTS Scavenging Ability

The ABTS scavenging assay was performed according to the method outlined by Boateng et al. [32]. To create the stock solution, a 7 mM solution of ABTS (10 mL) was added to 166 µL of potassium persulfate (K_2_S_2_O_8_) (140 mmol) and kept in dark at room temperature overnight. This mixture (1.2 mL) was mixed with DI water (60 mL) to prepare a solution, and the absorbance of the prepared solution was measured at 734 nm to ensure that it was in the range of 0.700 ± 0.004. Furthermore, 40 µL of the liquid extract or Trolox was mixed with 360 µL of this working solution and incubated at room temperature in dark for 10 min. The final solution was analysed with UV at a wavelength of 734 nm. The results were calculated in terms of mg of TE/g of pericarp.

#### 2.10.2. DPPH Scavenging Ability

The working solution for DPPH (1,2-diphenylpicrylhydrazyl) was prepared in the dark with 100 mL of methanol and 5 mg of DPPH. The mixture was let to react for 2 h and afterwards the absorbance was measured at 520 nm to check whether it was approximately 0.710 ± 0.0015. After confirmation, 400 µL of this solution was mixed with 100 µL of the liquid extract or Trolox and again incubated in dark at room temperature for 30 min, followed by measuring absorbance at 520 nm. The results were calculated in terms of mg of TE/g of pericarp [28].

#### 2.10.3. Cupric Ion Reducing Antioxidant Capacity (CUPRAC)

A working solution was prepared using equal proportions of DI water, 10 mM CuCl_2_, a 1 M solution of NH_4_Ac, and a 7.5 mM solution of neocuproine. This working solution (800 µL) was mixed with 20 µL of Trolox or the liquid extract and incubated for 60 min at 25 °C. As reported by [33], the mixture was analysed at a wavelength of 450 nm and the results are expressed in mg of TE/g of pericarp [20].

#### 2.10.4. Reducing Power (RP) Assay

The method described by Lim et al. [34] was followed, wherein 0.5 mL of a solution of potassium ferricyanide (1 mg/10 mL) was mixed with 0.5 mL of the liquid extract or Trolox. This was followed by the addition of 0.5 mL of 0.2 M phosphate buffer (pH 6.6) and an incubation at 50 °C for 20 min. A trichloroacetic acid solution (0.5 mL) having a concentration of 1 g/10 mL was mixed after the incubation, and the solution was centrifuged at 10,000 rpm for 10 min at a temperature of 4 °C. Finally, 0.5 mL of ferric chloride at a concentration of 1 mg/mL was mixed with 1 mL of this centrifuged mixture along with 1 mL of DI water. The mixture was vortexed and immediately observed under UV at a wavelength of 700 nm. The results are expressed in terms of mg of TE/g of pericarp.

### 2.11. Statistical Analysis

The screening and optimization experiments were designed using Minitab software Version 18 (Minitab Inc., State College, PA, USA). Tukey’s test was used for the analysis at *p* < 0.05. The 3D response surface plots, regression analysis, and perturbation plots were created using Design Expert Version 11 (Stat-Ease Inc., Minneapolis, MN, USA). All experiments were performed in triplicate, and the results of pairwise comparisons (a, b, and c) were calculated and presented as ±SDs (±standard deviations) from *n* = 3 repetitions.

## 3. Results and Discussion

### 3.1. Physicochemical Characteristics of DESs with Different Water Concentrations

Polarity, pH, and FTIR measurements of raw DES formulation without water and the formulations diluted with different concentrations of water were performed to observe any changes in the chemical bonds that might affect the extraction of polyphenolic compounds. In addition, an FTIR analysis was also performed on the individual constituents of the DES prior to mixture formulation. The addition of water in the DES-based solvent systems, to a certain extent, has been reported to increase phytochemical extraction due to improvements in certain physicochemical properties, such as polarity and surface tension, and by reducing the viscosity of the system. These changes increase solvent penetration into the cell membrane and accomplish better mass transfer rates, increasing the yield of extracted compounds [35]. However, if too much water is added to the system, it reduces the solvent efficiency by weakening the hydrogen bonds [36]. As shown in Table 1, the system polarity changed from 48.21 kcal/mol with no water to 48.43 kcal/mol with the addition of 10% water. However, the value was lower at 48.11 kcal/mol upon 50% water addition. In general, there were minor changes in the system polarity upon water addition, lacking any consistency. Similarly, the system pH also experienced a change with the addition of water, which can be explained by the resultant changes in the H^+^ ion concentrations. There was a slight increase in pH between when no water (pH 1.50) was present in the system and a 20% water concentration (pH 1.71); however, the pH value stabilized to approximately 1.65 upon 30, 40, and 50% water addition. It has been reported that the solvent pH may vary with changing temperatures, whereas, in this work, the pH for all the solvent systems was measured at room temperature and not during the extraction process [37].

**Figure 1:**
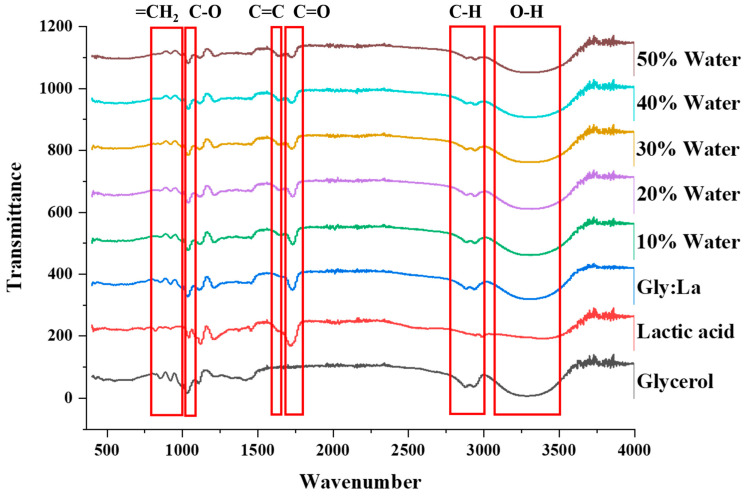
FTIR spectra of constituents and DESs formulated with different water concentrations.

Figure 1 shows the FTIR spectrum of the individual constituents, raw DES, and DES samples with different water concentrations. An FTIR analysis was initially performed to observe the chemical structure and type of bonds present in the DES, and later it was performed to track the bond stretching, shifting, and resulting changes due to water addition. Multiple functional groups were identified in the solvent systems, as shown in the Figure 1. A major dent in the spectra at 3200–3500 cm^−1^ was associated with the O-H stretching bond, closely followed by C-H stretching ranging from 2500 to 3000 cm^−1^. Furthermore, C=O stretching was observed at 1710 cm^−1^, and changes corresponding to C=C allyl stretching was identified in the range of 1615–1682 cm^−1^. A couple of other bonds identified in the IR spectrum were related to C-O at 1000–1112 cm^−1^, and =CH_2_ wagging vibrations were observed ranging from 727 to 1000 cm^−1^. These bonds were identified in accordance with the information made available by Sigma [38] and Rashid et al. [39]. In the case of lactic acid alone, minimal bond stretching was observed when it comes to the O-H and C-H bonds, whereas the stretching was more pronounced for glycerol, the second constituent of the DES. However, when both these constituents were combined to form the DES solution, the resultant mixtures demonstrated considerable stretching, as observed at wavelengths ranging between 3200–3500 cm^−1^ and 2500 to 3000 cm^−1^. These observations validated the formulation of the DES used for subsequent extraction studies. Further differences in peak intensities of the C=O, C-O, and =CH_2_ bonds can be observed with the changing concentrations of water in different DES mixtures. Overall, the IR spectral analysis confirmed the formation of the eutectic mixtures, implying that the requisite hydrogen bonds were formed, and an increased concentration of water did seem to have altered the chemical structures of the resulting DES combinations.

### 3.2. Screening of Parameters Using the Plackett–Burman Design (PBD)

**Figure 2:**
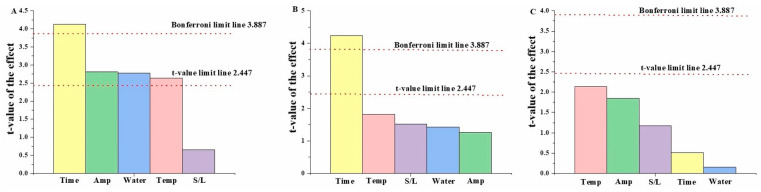
Pareto charts showing the influence of the screened factors on responses for total anthocyanins (**A**), total phenolics (**B**), and condensed tannins (**C**).

The Plackett–Burman design was selected to screen the significant parameters for subsequent optimization, as this design works on rational planning and statistical discrimination, wherein it generates maximal information from minimal experimentation, reducing the waste of time and energy [16]. When various factors come into play in an extraction-related experiment, some factors might be more significantly affecting the extraction yield compared to the others, and such behaviour can be identified by performing screening experiments prior to running the response surface design (RSM) [40]. The ultrasonication time (Time) (min), water bath temperature (Temp) (°C), concentration of water (Water) (%, *v*/*v*), ultrasonic amplitude (Amp) (%), solid-to-solvent ratio (S/L), and treatment time (time) were the parameters that were screened using the yields of TAC, TPC, and CT as responses, summarized in Table 2. A total of 13 screening experiments were conducted based on an analysis conducted using Minitab software (Minitab, LLC). Pareto charts showing the influence of specific factors on different responses are shown in Figure 2.

After the evaluation of the screening parameters, it was observed that the factors Time, Temp, Water, and Amp had significant *p*-values (Table 2). The t-values for the factors Water and S/L were positive in the case of TAC, and negative values were observed for Time, Temp, and Amp. For TPC, all the factors had positive t-values except for Time, and the *p*-value was significant for Time. In the case of CT, the factor Temp had a significant *p*-value; however, the t-values were negative for all the factors except for Water. Here, the t-value signifies the positive or negative effect of that factor on the response analysed.

Only the factor Water had a positive effect on all three responses (TAC, TPC, and CT). The factor S/L also demonstrated positive t-values for TAC and TPC, but the values were not significant enough to cross the t-value threshold on the Pareto chart (Figure 2). In other words, the t-value shows the significance of the effect of a particular factor on the corresponding response. The higher the magnitude of the t-value, the higher is the effect on the response. The factor Water showed a positive effect on all responses, which can be explained based on multiple reasons. Firstly, the viscosity of the solvent system decreased due to the addition of water, which is one of the confounding factors affecting extractions involving DESs. Reduced viscosity also results in an increased mass transfer that ultimately helps with the greater removal of polyphenols from the cell membranes to the solvent [41]. The addition of water to the DES increases the formation of hydrogen bonds with the compounds in the solvent, thus enhancing the extraction of analytes present in the mix. Another explanation could be attributed to the fact that a change in the polarity of the DES upon the addition of water enhances the extraction of polyphenolic compounds [42]. Furthermore, the factor Temp showed a variable trend in t-values, where it was positive for TPC and negative for TAC and CT, implying an enhanced extraction of TPC at higher temperatures but decreasing recovery of TAC and CT. Although increasing the temperature was observed to enhance the extraction of all three responses when an ethanol-based solvent was used in a previous study, this enhanced extraction was observed up to a certain limit and afterwards a steady degradation of the polyphenols was reported [16]. When comparing the observed t-values from this study with the previous work, similar trends were identified, where TPC showed higher t-value for temperature compared to the TAC and CT. Therefore, temperature demonstrates a varying effect on polyphenol extraction from plant cell membranes, and the application of higher extraction temperatures in combination with ultrasonic power may degrade the anthocyanins and condensed tannins, resulting in a decreased extraction efficiency [23].

As was the case with temperature, the factor Amp showed similar trends of a positive effect on TPC and negative effects on TAC and CT extraction. The ultrasonic treatment time (Time) was observed to significantly affect all the responses in a negative manner, which could be attributed to the fact that increased cavitation over time increases the overall system temperature, resulting in the thermal degradation of anthocyanins, phenolic acids, and tannins. However, this does not necessarily mean that time is not an influencing factor; in fact, an increased ultrasonication time helps promote higher phytochemical extraction to a certain limit. However, a further increase in the ultrasonication time results in the degradation of polyphenols when combined with other influencing factors, such as temperature and ultrasound amplitude [23,43]. Finally, the effect of S/L was not observed to be significant enough to cross the t-value limit line (2.447) in the Pareto chart for all three responses; however, it displayed positive influences on TAC and TPC and a negative effect on CT. At a lower S/L, the mixture becomes highly viscous, whereas at a higher S/L, it becomes too dilute. Cavitation increases at a higher S/L, resulting in an increased mass transfer from the biological material to the solvent. Due to the insignificant effect of S/L, an optimal S/L level identified from preliminary experiments was selected for the response surface optimization. The observed results were mostly in accordance with previously reported studies, and as was the case in those experiments, it was decided to conduct further experiments with the identified optimal S/L ratio [16,23].

Finally, a regression analysis was performed to test the variability or closeness of the predicted values to the actual values. The results of this analysis were ascertained based on the coefficients of determination or R^2^ values. The R^2^ values for TAC, TPC, and CT were 87.36, 82.59, and 62.09%, respectively. From these values, it could be deduced that the model was robust enough for TAC and TPC, but was not so strong for CT.

### 3.3. Response Surface Optimization (RSM) Using the Box–Behnken Design (BBD)

#### 3.3.1. Model Fitting

From the initial screening experiment, four significant parameters were identified (Time, Temp, Water, and Amp), and used for extraction optimization following the Box–Behnken design with the predicting parameters being TAC, TPC, and CT concentrations. As shown in Table 3, the maximum TAC observed was 37.79 g of C3G/kg of pericarp and the lowest was 28.97 g C3G/kg of pericarp. In the case of TPC, the highest and lowest values were 125.79 and 95.00 g of GAE/kg of pericarp, respectively. The obtained CT values were highest at 245.30 g of EE/kg of pericarp and lowest at 191.00 g of EE/kg of pericarp. These results were comparable to the reported amounts of polyphenolic compounds extracted from the purple corn pericarp using acidified organic solvents in an earlier work [16].

Therefore, the overall effectiveness of the DES for extracting bioactive compounds from purple corn pericarp was demonstrated. As shown in Table 4, underlying statistical parameters were calculated for the responses and the coefficient of variation (CV) values were 6.53, 9.01, and 6.56% for TAC, TPC, and CT, respectively. CV is used as a standard for measuring the variability when the magnitude of responses is different, and it serves to eradicate the standard deviation (SD) as a factor in the overall variability [44]. Upon further analysis using ANOVA, it was revealed that the response of TAC was significantly influenced by the individual factors Temp and Amp, whereas TPC and CT were not influenced by any of the individual, squared, or 2-way interaction factors. The uncoded models for TAC, TPC, and CT are presented in Table 4.

Following the regression analysis of all the individual variables, the R^2^, adjusted R^2^, and predicted R^2^ were calculated, as shown in Table 4. The R^2^ value, which shows how well the model would fit any particular response, was 86.26% for TAC, which is quite significant and proved that the model would fit TAC. However, TPC and CT had lower R^2^ values of 39.80 and 39.47%, respectively. The adjusted R^2^ and predicted R^2^ for TAC were 70.24 and 28.74%, and these values were null for the other two responses. Observing the lack of fit gave some interesting insights, as the model fitted TAC and CT with values of 0.589 and 0.376, correspondingly, but it was significant for TPC, with a lack of fit of 0.012, which means that the null hypothesis of the model not fitting was true for TPC but false for TAC and CT. Thus, the model was deemed to be working for TAC and CT extraction. Since TAC was the only response that had significant influencing factors and a satisfactory R^2^ of 86.26%, we further analysed and presented the resulting surface plots for TAC.

#### 3.3.2. Impacts of the Extraction Parameters on Polyphenolic Recovery During Optimization

The performance measure, also called the response, is influenced by multiple factors, whether it be the ultrasonic amplitude, extraction temperature, extraction time, amount of water added to the DES, or synergistic effects of all. RSM is utilized to develop the optimal extraction conditions using statistical modelling to meet the desired extraction response. The interactions between independent variables are controlled and modelled to the best-fitting response [16,45]. To extract the maximum quantity of polyphenols based on various ultrasonic extraction factors and DES combinations, the most efficient model was determined using various response surface plots, extraction responses, and perturbation plots for TAC. Similar plots were generated for TPC and CT. However, since no significant differences were observed, this resulted in uniform planes in the response surface plots (Supplementary Figures S1 and S2).

##### Impact of Time on Polyphenol Extraction

**Figure 3:**
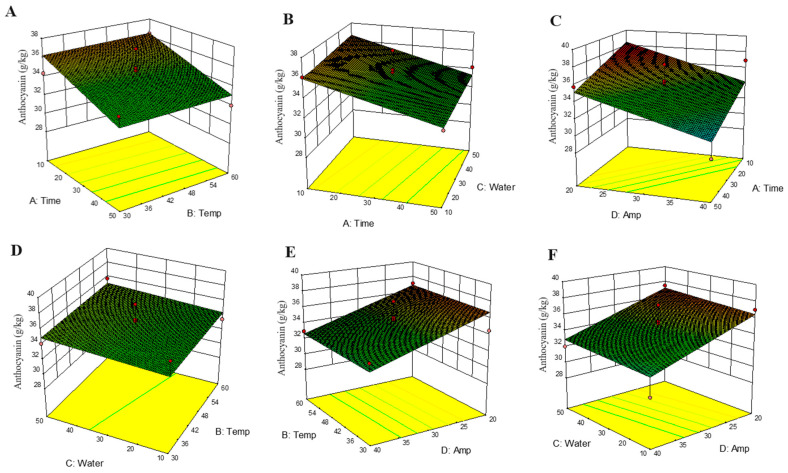
(**A**–**F**) Response surface plots for the effects of interactions between Time, Temp, Water, Amp, and the S/L ratio on TAC extraction by combining DES with UAE. The red dots indicate the design points above the predicted values and the yellow dots indicate the design points below the predicted values.

Time is a significant factor affecting the extraction of polyphenols from biological material. In this study, time was used as a factor and its influence was observed on the extraction of TAC, TPC, and CT. Time also influences other extraction factors, including temperature, which increases with repetitive cavitation and in turn facilitates cell wall disruption and the production of micro-bubbles during the compression and rarefaction cycles caused due to the ultrasonic waves. This in turn increases the penetration of the solvent into the cell wall, resulting in improved extraction [16,46]. It can be noted from Figure 3 that with an increased time of ultrasound treatment, the quantity of anthocyanins extracted decreased. Due to the interactions between temperature, water, and amplitude, a shorter time tends to extract the maximum quantity of anthocyanins. Degradation of anthocyanins with increased extraction times can be because of the individual effects of time and amplitude or due to a synergistic effect of multiple factors during the extraction process [47]. A linear trend has been observed in several other studies, where increasing the extraction times decreased the anthocyanin yield for mango polyphenols [48], as well as for the blueberries and cherries [49]. Boateng et al. [16] also reported a similar effect while extracting polyphenolic compounds from purple corn pericarp.

##### Impact of Temperature on Polyphenol Extraction

Polyphenols constitute multiple compounds belonging to a diverse family of anthocyanins, tannins and phenolics. Each individual compound differs and may have a tendency to be extracted in a uniquely optimized manner [16]. Temperature is known to be a significant factor influencing the extraction of polyphenols from food matrices and was evaluated in this work. However, after the performing the analysis using ANOVA (Table 4) and going through the response surface plots in Figure 3, temperature was observed to be a non-significant factor for the extraction of TAC, TPC, and CT in this study. In a previous study on the extraction of purple corn pericarp phytochemicals using an ethanol-based solvent, it was observed that increased temperatures did not affect the TAC yield, but they enhanced the extraction of TPC and CT. The temperature treatments evaluated in this study were 30 °C (−1), 45 °C (0), and 60 °C (+1). Polyphenol extraction from plant material is reported to increase until the temperatures increases to a certain level due to the softening of plant tissue, and disruptions affecting the association between polyphenolic compounds and proteins or carbohydrates [50]. However, anthocyanin degradation is reported to start at temperatures above 60 °C, and the half-life of C3G at this temperature is 16.7 h. Higher temperatures enhance extraction efficiencies by increasing the diffusion coefficient and increasing the compound’s solubility in the solvent. Hence, higher temperatures are not always detrimental but there needs to be an optimal point where the temperature helps with the extraction of these compounds without negatively affecting their stability in the solvent [51].

##### Impact of the Ultrasound Amplitude on Polyphenol Extraction

The ultrasound amplitude or ultrasound intensity is used to report the ultrasonic power being used by the equipment and has a direct bearing on the ultrasonic cavitation taking place in the medium. Ideally, with an increasing amplitude, the breakdown of cell membrane increases, and this results in an efficient release of polyphenols. Increased ultrasonic power increases the cavitation effect, eventually increasing the system pressure due to the rapid expansion and compression of the bubbles formed [16]. However, when the combined effects of amplitude with other factors such as time, temperature and water concentration were evaluated, all these factors were observed to extract the highest concentration of polyphenols at the lowest ultrasound amplitude, as shown in Figure 3. In the graph showing the effects of time and amplitude on TAC, the lowest values of both factors yielded the highest amount of TAC. Increasing temperatures did not affect the extraction of TAC, as the yield was observed to be similar for all temperature levels at the lowest amplitude. At a 20% amplitude, the TAC yield decreased with decreasing water concentration. At the same time, TPC and CT did not experience any effect of amplitude, either individually or in a linear or quadratic manner.

##### Impact of the Water Concentration on Polyphenol Extraction

As observed in Figure 3, water did have an effect on the extraction of TAC, whereas no effect of water was observed on TPC and CT. The addition of water to the DES formulations makes them less viscous and helps increase the mass transfer rates during extraction [40]. The extraction efficiency of bioactive compounds from date palm depended upon the concentration of water in the DES system [52]. Anthocyanins are water-soluble compounds and less soluble in non-polar solvents. When the water concentration was increased to 10%, the solvent polarity increased to 48.43 kcal/mol; however, any subsequent addition of water did not result in an increased polarity, and the value was 48.11 kcal/mol at a 50% water concentration (Table 1). The addition of water resulted in decreased DES viscosity, and in previous experiments, a water-diluted DES resulted in greater polyphenol extraction compared to an undiluted, viscous DES system. Therefore, an increase in the water concentration to a certain degree reduces the viscosity of the DES, making the transfer of bioactive compounds in the solvent easier. However, when too much water is added, such as at a 50% concentration, the polarity is reduced, resulting in the decreased extraction of compounds because the interaction between the target molecule and DES is significantly weakened [53].

#### 3.3.3. Optimization and Verification of the Predictive Model

Optimized parameters were obtained using the response optimizer function in Minitab software. The obtained conditions were as follows: 10 min of ultrasonication time, a water bath temperature of 60 °C, water concentration of 42.73%, and 40% ultrasound amplitude. The predicted fit response values were 35.62 g of C3G/kg, 122.21 g of EEs/kg, and 245.51 g of GAEs/kg pericarp for TAC, TPC, and CT, respectively (Table 5). The desirability function (DF) was observed for the Harrington scale-based optimization, which indicates the overall desirability of the model. The given desirability was 0.858, as shown in Table 4. The acceptable scale for this desirability function lies between 0.8 and 1, which means that the obtained optimized values could be depended upon. Validation tests were run in triplicate to observe any deviation in the obtained and experimental results. The experimental yields obtained were 36.31 g of C3G/kg, 103.16 g of EEs/kg, and 237.54 g of GAEs/kg pericarp for TAC, TPC, and CT, respectively (Table 5). The relative standard error (RSE) was calculated to check the model’s consistency, and it was observed that the RSE among the predicted and observed values was less than 10%, which was acceptable, and the model was confirmed to be dependable.

### 3.4. The Quality of the Optimized Polyphenolic Extract

After the final optimization and verification, the extract prepared using the optimized parameters was evaluated for the total flavonoid content (TFC) and its antioxidant properties by employing four different assays (ABTS, DPPH, CUPRAC, and reducing power). As reported previously, every distinct antioxidant assay has its associated advantages and disadvantages, and no one assay can be a reliable determinant of the total antioxidant capacity of a particular biological material [20]. It is also an established fact that the antioxidant properties of a biological material detected by different in vitro assays might not be truly representative of those activities in human beings. The results of these methods are reported as total antioxidant activity, and they work on the basic principle of eradicating free radicles by a solution being tested. There are three underlying mechanisms associated with such assays, including the chelation of transition metals, reaction or hydrogen atom-based assays, and single electron transfer (SET) [54]. An antisolvent method was used to remove the polyphenolic compounds from the DES solvent after extraction. After extraction, water was added to the DES extracts to make it easy to recover the polyphenolic compounds. This method of phytochemical recovery has been reported by Huang et al. [55], wherein they sedimented rutin from a DES extract by adding water. Rutin is less soluble in water but has a higher solubility in DES, and the authors precipitated rutin by cooling the mixture to 0 °C followed by centrifugation. In an another study by Nam et al. [56], water was utilized as an antisolvent to remove flavonoids. Solid-phase extractions were reported to have performed better compared to the antisolvent method. In previous research, water addition facilitated better handling of the DES post-extraction since the DES extracts had higher viscosities without water addition, making it difficult to filter out the polyphenols from the substrate after centrifugation.

Another benefit derived from this method was an increased rate of mass transfer, which enhanced the extraction efficiency. As shown in Table 6, the amount of TFC present in the optimized extract was 40.80 ± 1.36 g of CEs/kg of pericarp. The antioxidant activities of the optimized extract for various assays ranged between a high of 351.59 ± 17.86 mg TE/g of pericarp for the CUPRAC assay to a low of 88.43 ± 1.6 mg of TE/g of pericarp for the DPPH assay. The antioxidant activity observed for the ABTS assay was 141.92 ± 10.29 mg of TE/g of pericarp, whereas the value was 199.89 ± 4.26 mg of TE/g of pericarp for the RP assay (Table 6). The antioxidant activities observed in polyphenolic extracts after optimization were similar to those reported by Kumar et al. [57] for CUPRAC and DPPH assays; however, the activity observed for the ABTS assay was more than fourfold higher. The biological activities of polyphenolic extracts from other natural substrates have been reported to be as follows: black soybeans demonstrated an ABTS activity of 4.97 mg of TE and DPPH activity of 0.42 mg of AAE per g of soybeans [58], whereas purple corn cob extract demonstrated 8.70 mg of TE of DPPH activity and 45.30 mg of TE of ABTS activity per g of cob powder [59]. Furthermore, 11.32 µmol of TE of DPPH activity and 38.87 µmol of TE of ABTS activity per g of corn were observed by Bhushan et al. [60]. Compared to the reported values of antioxidant activities in other studies, the purple corn pericarp extracts obtained by combining the DES with the ultrasound-assisted extraction in this study demonstrated greater antioxidant activities and can potentially serve as effective antioxidant agents in various applications.

### 3.5. HPLC-Based Phytochemical Profiling of the Optimized Extract

**Figure 4:**
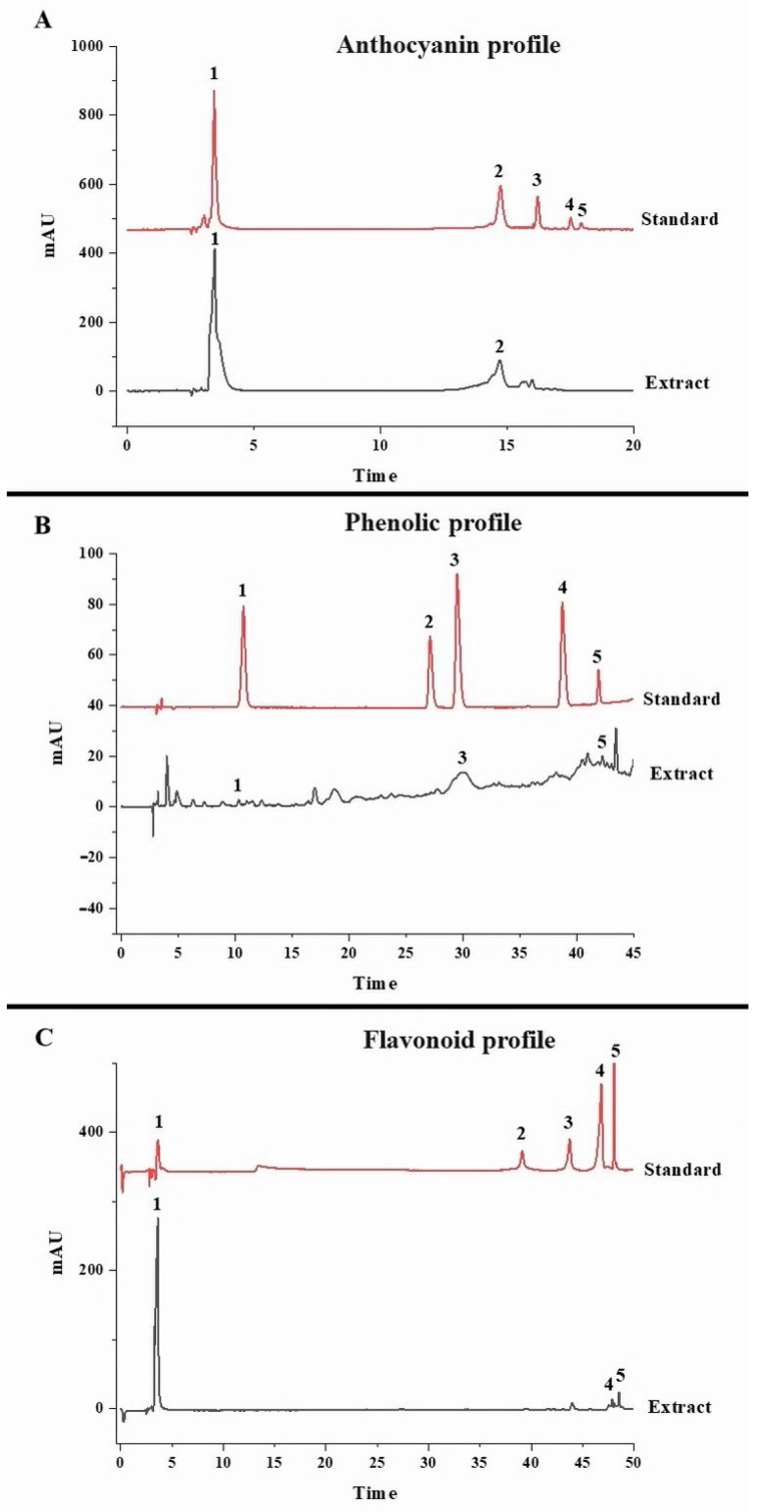
HPLC chromatograms for used standards and identifying anthocyanin (**A**), phenolic (**B**) and flavonoid (**C**) compounds in the optimized extract. Note: Standard peaks identified in the anthocyanin profile belong to (1) cyanidin-3-glucoside, (2) delphinidin, (3) cyanidin chloride, (4) peonidin, (5) malvidin, and (6) pelargonidin chloride. Standard peaks identified in the phenolic profile belong to (1) gallic acid, (2) chlorogenic acid, (3) caffeic acid, (4) ferulic acid, and (5) hesperidin. Standard peaks identified in the flavonoid profile belong to (1) epicatechin, (2) morin, (3) naringin, (4) quercetin, and (5) kaempferol.

After extracting the polyphenolic compounds utilizing the optimized and verified parameters, the samples were analysed with HPLC equipment using three different protocols for identifying and quantifying the distinct forms of anthocyanins, phenolic acids, and flavonoids. A total of eight unique compounds were detected: two of the identified compounds were anthocyanins, three were phenolic acids, and the remaining three belonged to the class of flavonoids (Figure 4). C3G and delphinidin were the detected anthocyanin forms, and these two are considered as the most abundant anthocyanin compounds present in a majority of coloured plant matrices. The concentration of C3G was 15.61 ± 0.49 mg/g of pericarp, whereas 16.25 ± 0.28 mg delphinidin/g of pericarp was quantified (Table 7). In several other studies, similar abundances of C3G and delphinidin have been reported in purple corn [20,61,62], black soybeans [58], and blueberries [63]. Out of a total of five phenolic acid standards used in this work, three were detected in the optimized extract, their concentrations being 0.70 ± 0.00 of mg of gallic acid/g of pericarp, 14.97 ± 0.18 mg of caffeic acid/g of pericarp, and 7.42 ± 0.80 mg of hesperidin/g of pericarp. These compounds have been reported to be the major phenolic acids present in different coloured corn varieties. Other research groups have reported similar phenolic acid profiles in dark-coloured grapes [64], as well as in strawberries and blueberries [65]. A higher concentration of phenolic acids is one of the major factors that contributes to the antioxidant properties of such extracts. Phenolic compounds tend to lower oxidative stress and are used as functional ingredients in various food and health supplements [16,20,57]. Other good sources of phenolic acids include mango, coffee, and banana, which have been reported to contain a similar phenolic acid profile to that identified in purple corn in the current study [66,67]. In terms of the flavonoid contents, 102.73 ± 1.66, 1.38 ± 0.99, and 0.37 ± 0.00 mg of epicatechin, quercetin, and kaempferol/g of pericarp, respectively, were detected (Table 7).

These very compounds were also detected in whole purple corn kernels in a previous study [20]. However, in an another study where various DES combinations were used to extract polyphenols from purple corn pericarp, only epicatechin and kaempferol were quantified [57]. In addition to the above two, quercetin was the third flavonoid quantified in this study. This could be attributed to the addition of water, which may have facilitated the extraction of quercetin, or this additional extraction may have been a result of the extraction optimization process. Furthermore, one-way ANOVA was performed on the HPLC data, and it was observed that epicatechin was the most abundant polyphenol extracted, followed by C3G and caffeic acid. Hesperidin and all the remaining compounds were grouped together. In a previous extraction optimization study using an ethanol-based solvent to extract polyphenols from purple corn pericarp, epicatechin was not detected [16]. However, the use of the DES may have facilitated relatively higher amounts of epicatechin being quantified in this study (102.73 mg/g pericarp). Similar flavonoid profiles of other coloured corn varieties have been reported by earlier researchers [67].

### 3.6. Comparison of the Metabolomic Profiles of the Optimized Extract and an Aqueous Extract

**Figure 5:**
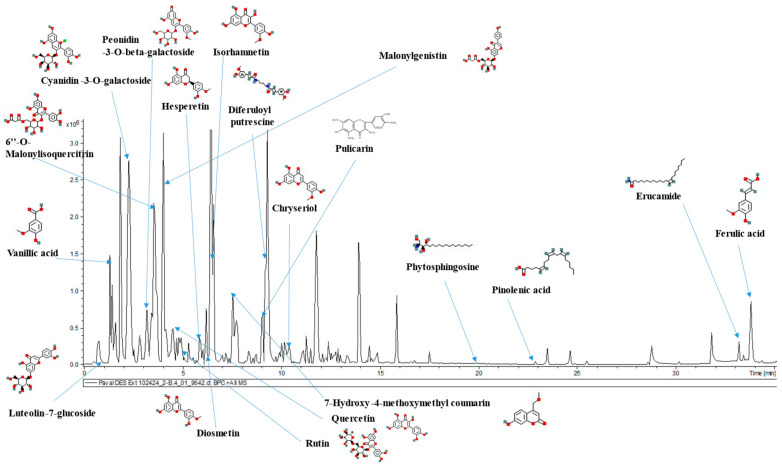
UHPLC-MS chromatogram for the optimized DES extract, identifying some of the bioactive compounds.

**Figure 6:**
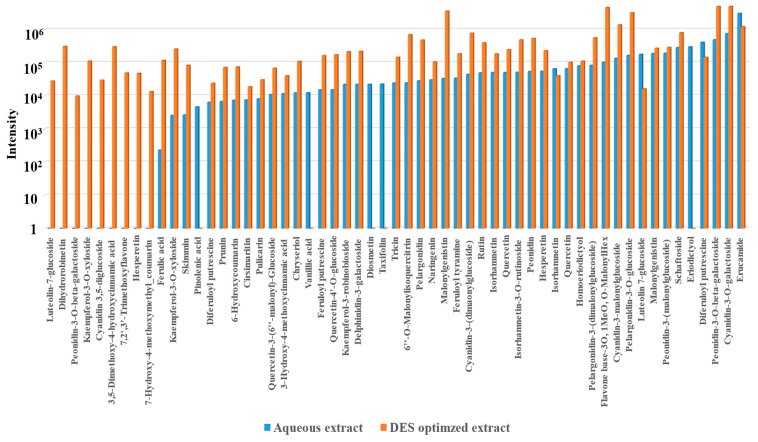
Comparisons of the intensities of bioactive compounds detected in both the aqueous and DES extracts during the metabolomic analysis.

Several researchers have reported the presence of multiple secondary plant metabolites in coloured corn varieties. The main aim for conducting this analysis was to identify whether there were any differences when it comes to the extracted bioactive compounds in the optimized DES extract and a plain aqueous extract. Since fruits and vegetables are composed of highly complex and unique matrices, different extraction methods and solvents result in the extraction of different types and varying quantities of phytochemical compounds [68,69]. Lao et al. [70] identified cyanidin, catechin-(4,8)-cyanidin-3,5-diglucoside, pelargonidin-3-glucoside, peonidin-3-glucoside, cyanidin-3-(6″-malonylglucoside), peonidin-3-(6″-malonylglucoside), pelargonidin-3-dimalonylglucoside, and other derivatives in extracts from various coloured corn cobs. In an another work by Lee et al. [71], the authors detected luteolin, cyanidin-3-O-glucoside, 6-gingerol, glycocholic acid, and linoleic acid in Malaysian purple corn extracted using an acidified ethanolic solvent. Furthermore, Peniche et al. [72] reported that cyanidin-based compounds and their derivatives were predominant in purple corn compared to the pelargonidin-based compounds and their derivates that were found to be abundant in red corn varieties extracted using a methanol-based solvent. Compared to the abovementioned studies, the optimized DES and UAE method extracted cyanidin, delphinidin, peonidin, pelargonidin, and their derivatives for anthocyanins, whereas the various phenolic acids detected included diferuloyl putrescine, ferulic acid, feruloyl putrescine, and vanillic acid (Table 8). The same phenolic acids have been reported to be present in corn tassels by Al-Khayri et al. [73]. Flavonoids were the most dominant class of compounds detected among the polyphenolic compounds that were present in the extracts. A total of 57 secondary plant metabolites, including 32 flavonoids, 12 anthocyanins, and 10 phenolic acids, were detected in the DES extract. For better identification of compounds extracted with DES, a labelled chromatogram with the major compounds identified along with their structures is presented in Figure 5. When it comes to the comparison between the optimized DES extract and the aqueous extract, nine additional polyphenol compounds were quantified in the optimized DES extract that were not present in the water-based solvent. These included two anthocyanins (peonidin and cyanidin derivative), two phenolic acids (7-hydroxy-4-ethoxymethylcoumarin, and 3,5-dimethoxy-4-hydroxycinnamic acid), and the rest were flavonoid compounds. The presence or absence of a specific compound in the extracts is depicted with a corresponding positive or a negative sign in Table 8. Furthermore, the intensities of the compounds detected in DES and aqueous extracts during the metabolomic analysis were compared to observe any differences (Figure 6). In general, the intensities were observed to be greater in case of the metabolites present in the DES extract, implying a greater extraction efficiency compared to the aqueous extract.

## 4. Conclusions

For the first time, advanced statistical techniques were employed to identify the optimal polyphenol extraction parameters from the purple corn pericarp waste stream by combining a deep eutectic solvent (DES) and ultrasound-assisted extraction (UAE) technique. The DES was formulated using a previously reported method and characterized using FTIR, pH, and polarity measurements. The FTIR analysis revealed shifts in bonds before and after the formation of DES. Plackett–Burman design was used to screen five explanatory variables, namely, time (Time), temperature (Temp), water concentration (Water), amplitude (Amp), and solid-to–liquid ratio (S/L). the total anthocyanin concentration (TAC), total polyphenolic concentration (TPC), and condensed tannin (CT) concentration were the response variables. After the identification of four significant factors, the Box–Behnken design was utilized in an RSM-based approach to identify the optimal extraction parameters. A linear trend was observed in the extraction of TAC, whereas there were no differences in TPC and CT recoveries for different variable combinations. The lack of fit for TAC and CT concentrations was 0.589 and 0.376, respectively, which was significant, while TPC had an insignificant lack of fit (0.012). The optimal extraction parameters were identified to be time (10 min), temperature (60 °C), water concentration (42.73%), and amplitude (40%). Under these conditions, the extracted TAC was 36.31 ± 1.54 g of cyanidin-3-glucoside (C3G)/kg of pericarp, 103.16 ± 6.17 g of gallic acid (GA)/kg of pericarp, and 237.54 ± 9.98 g of epicatechin (EE)/kg of pericarp, with an acceptable desirability index of 0.858. The relative standard errors among the predicted and experimental yields of TAC, TPC, and CT were less than 10%, which was acceptable, and the developed model was confirmed to be dependable. HPLC quantification revealed that C3G, delphinidin, gallic acid, caffeic acid, hesperidin, epicatechin, quercetin, and kaempferol were the most abundant compounds present in the extract. Significant antioxidant activities were observed in the recovered extract through ABTS, DPPH, CUPRAC, and RP assays. The DES extracted compounds that do not have an affinity for water-based solvents, and, in general, the magnitude of the extracted compounds was higher in the DES. A total of 57 secondary plant metabolites, including 12 anthocyanins, 10 phenolic acids, and 32 flavonoids, were identified through UHPL-MS analysis. The UAE technique combined with the DES can efficiently extract polyphenols from purple corn pericarp in significantly less time. The proposed extraction technique can be an efficient and economically viable alternative to other extraction methods, facilitating time and energy savings.

## Figures and Tables

**Table 1:** Physicochemical characteristics of DESs with different water concentrations.

Water (%, *v*/*v*)	Polarity (kcal/mol)	pH
0	48.21 ± 0.00 bc	1.50 ± 0.02
10	48.43 ± 0.13 a	1.64 ± 0.01
20	48.30 ± 0.00 ab	1.71 ± 0.01
30	48.13 ± 0.00 c	1.65 ± 0.01
40	48.11 ± 0.05 c	1.65 ± 0.02
50	48.11 ± 0.05 c	1.66 ± 0.01

Means ± standard deviations. Homogenous groups were determined from three repetitions (*n* = 3). Means followed by the same letter in a column are not different (*p* < 0.05).

**Table 2:** Screening experiment using showing the Plackett–Burman design (PBD) using various extraction variables and the experimental data for polyphenols from purple corn pericarp.

	Variables					Responses		
Run Order	Time	Temp	Water	Amp	S/L	TAC	TPC	CT
1	50	30	50	20	20	33.23 ± 0.17	87.4 ± 1.78	230.84 ± 7.28
2	50	60	10	40	20	18.01 ± 0.63	89.74 ± 2.02	172.93 ± 11.03
3	10	60	50	20	60	36.62 ± 0.60	141.43 ± 2.46	181.06 ± 7.94
4	50	30	50	40	20	31.37 ± 0.46	90.89 ± 0.38	225.09 ± 6.42
5	50	60	10	40	60	18.52 ± 0.39	106.83 ± 0.24	131.37 ± 8.07
6	50	60	50	20	60	35.84 ± 0.67	109.00 ± 3.52	211.51 ± 13.27
7	10	60	50	40	20	35.48 ± 1.16	166.43 ± 6.91	225.69 ± 6.42
8	10	30	50	40	60	32.02 ± 0.04	144.65 ± 18.61	197.18 ± 9.26
9	10	30	10	40	60	32.76 ± 0.41	136.68 ± 5.66	216.08 ± 8.41
10	50	30	10	20	60	31.73 ± 0.40	103.57 ± 0.16	265.66 ± 4.69
11	10	60	10	20	20	32.65 ± 0.78	136.82 ± 2.27	230.48 ± 13.71
12	10	30	10	20	20	35.24 ± 0.43	89.34 ± 4.66	238.98 ± 18.23
13	30	45	30	30	40	34.84 ± 0.22	98.59 ± 1.26	223.51 ± 2.56
ANOVA	TAC		TPC		CT			
	t-effect	*p*-value	t-effect	*p*-value	t-effect	*p*-value		
Time	−4.13	0.006	−4.24	0.005	−0.51	0.631		
Temp	−2.64	0.039	1.82	0.119	−2.14	0.076		
Water	2.78	0.032	1.43	0.203	0.15	0.883		
Amp	−2.81	0.031	1.26	0.255	−1.84	0.115		
S/L	0.66	0.535	1.52	0.180	−1.18	0.284		
Regression model								
TAC = 46.17 − 0.1821 Time − 0.1552 Temp + 0.1225 Water − 0.2478 Amp + 0.0290 S/L + 3.72 Ct × Pt								
TPC = 80.9 − 0.950 Time + 0.543 Temp + 0.320 Water + 0.564 Amp + 0.340 S/L − 18.3 Ct × Pt								
CT = 338.0 − 0.217 Time − 1.227 Temp + 0.066 Water − 1.585 Amp − 0.505 S/L + 12.9 Ct × Pt								

Means ± standard deviations from three replicates (*n* = 3).

**Table 3:** Experimental data for polyphenol extraction from purple corn pericarp using the Box–Behnken design (BBD).

Run Order	Time	Temp	Water	Amp	TAC	TPC	CT
1	10	30	30	30	34.36 ± 1.29	119.62 ± 5.60	212.20 ± 10.97
2	50	30	30	30	33.99 ± 0.87	125.79 ± 14.40	204.40 ± 20.22
3	10	60	30	30	36.36 ± 1.39	121.43 ± 10.04	213.64 ± 11.06
4	50	60	30	30	32.01 ± 2.09	120.08 ± 7.17	200.25 ± 13.91
5	30	45	10	20	36.98 ± 2.57	109.26 ± 2.80	217.28 ± 9.65
6	30	45	50	20	37.45 ± 2.70	117.51 ± 8.20	205.30 ± 14.62
7	30	45	10	40	29.91 ± 1.57	120.42 ± 20.27	191.00 ± 12.65
8	30	45	50	40	32.00 ± 1.07	107.28 ± 4.86	226.38 ± 10.25
9	10	45	30	20	37.79 ± 0.56	95.00 ± 1.94	233.68 ± 13.37
10	50	45	30	20	35.74 ± 1.80	127.42 ± 18.68	208.87 ± 12.69
11	10	45	30	40	36.91 ± 1.28	100.08 ± 3.68	233.68 ± 8.76
12	50	45	30	40	28.97 ± 1.10	105.29 ± 4.60	216.24 ± 6.43
13	30	30	10	30	36.14 ± 1.03	100.92 ± 6.56	244.64 ± 17.14
14	30	60	10	30	33.46 ± 0.33	104.68 ± 1.98	229.18 ± 10.16
15	30	30	50	30	34.15 ± 1.07	97.03 ± 1.94	231.24 ± 7.52
16	30	60	50	30	35.74 ± 0.65	111.97 ± 7.52	241.14 ± 12.66
17	10	45	10	30	36.09 ± 2.06	98.19 ± 3.20	245.30 ± 7.98
18	50	45	10	30	32.41 ± 1.69	102.65 ± 8.61	225.05 ± 8.79
19	10	45	50	30	35.33 ± 1.02	97.69 ± 1.80	219.44 ± 9.84
20	50	45	50	30	34.07 ± 1.21	102.95 ± 5.08	210.89 ± 9.00
21	30	30	30	20	34.50 ± 2.47	96.79 ± 3.94	233.72 ± 7.64
22	30	60	30	20	37.10 ± 0.85	103.14 ± 3.08	237.68 ± 11.85
23	30	30	30	40	33.39 ± 1.50	101.21 ± 7.87	223.68 ± 7.78
24	30	60	30	40	32.95 ± 1.94	114.56 ± 11.11	241.09 ± 7.63
25	30	45	30	30	35.07 ± 1.02	102.12 ± 11.69	210.06 ± 9.11
26	30	45	30	30	35.47 ± 3.18	103.20 ± 5.65	210.70 ± 2.41
27	30	45	30	30	37.29 ± 1.75	100.50 ± 5.87	231.60 ± 9.45

Means ± standard deviations from three replicates (*n* = 3).

**Table 4:** ANOVA of RSM and regression analysis of the BBD for extracting polyphenolic compounds from purple corn pericarp.

	TAC		TPC		CT	
Source	F-Value	*p*-Value	F-Value	*p*-Value	F-Value	*p*-Value
Model	5.38	0.003	0.57	0.845	0.56	0.851
Linear	14.42	0	0.67	0.628	0.68	0.622
Time	21.36	0.001	1.85	0.199	2.55	0.137
Temp	0.06	0.803	0.81	0.386	0.05	0.824
Water	0.94	0.35	0	0.966	0.10	0.760
Amp	35.3	0	0	0.994	0.01	0.940
Square	0.91	0.49	0.59	0.677	0.54	0.708
Time × Time	1.60	0.23	1.17	0.301	0.24	0.635
Temp × Temp	1.78	0.207	1.23	0.290	1.12	0.312
Water × Water	1.78	0.206	0.01	0.915	0.37	0.556
Amp × Amp	2.08	0.175	0.37	0.557	0.08	0.776
2-Way Interaction	2.34	0.099	0.49	0.807	0.49	0.802
Time × Temp	2.59	0.133	0.12	0.740	0.03	0.870
Time × Water	0.86	0.372	0	0.972	0.12	0.732
Time × Amp	5.68	0.035	1.51	0.243	0.05	0.829
Temp × Water	2.99	0.110	0.25	0.623	0.58	0.462
Temp × Amp	1.51	0.242	0.10	0.758	0.16	0.694
Water × Amp	0.43	0.524	0.93	0.353	2.01	0.181
Lack-of-fit	0.589		0.012		0.376	
Mean	34.66		107.66		222.16	
SD	2.27		9.70		14.57	
CV (%)	6.53%		9.01%		6.56%	
R^2^	86.26%		39.80%		39.47%	
Ad R^2^	70.24%		0.00%		0.00%	
Pred R^2^	28.74%		0.00%		0.00%	
Desirability index =	0.858					
**Regression equations**						
TAC = 17.5 + 0.346 Time + 0.436 Temp − 0.139 Water + 0.639 Amp − 0.00169 Time × Time − 0.00317 Temp × Temp − 0.00179 Water × Water − 0.00771 Amp × Amp − 0.00332 Time × Temp + 0.00143 Time × Water − 0.00736 Time × Amp + 0.00356 Temp × Water − 0.00507 Temp × Amp + 0.00202 Water × Amp						
TPC = 137 + 0.73 Time − 2.37 Temp + 0.44 Water − 0.44 Amp + 0.0130 Time × Time + 0.0236 Temp × Temp − 0.0013 Water × Water + 0.0290 Amp × Amp − 0.0063 Time × Temp + 0.0005 Time × Water − 0.0340 Time × Amp + 0.0093 Temp × Water + 0.0117 Temp × Amp − 0.0268 Water × Amp						
CT = 439 − 0.14 Time − 4.15 Temp − 3.68 Water − 4.36 Amp − 0.0088 Time × Time + 0.0339 Temp × Temp + 0.0109 Water × Water + 0.0210 Amp × Amp − 0.0047 Time × Temp + 0.0073 Time × Water + 0.0092 Time × Amp + 0.0211 Temp × Water + 0.0224 Temp × Amp + 0.0592 Water × Amp						

**Table 5:** Optimization and verification of the developed model.

Name	Aim	LL	UL	Optimum	Verification	RSE (%)
Time (min)	In range	10	50	10	10	
Temp (°C)	In range	30	60	60	60	
Water (% *v*/*v*)	In range	10	50	42.73	42.73	
Amplitude (%)	In range	20	40	40	40	
TAC	Maximize	28.97	37.79	35.62	36.31 ± 1.54	1.41
TPC	Maximize	95.00	127.42	122.21	103.16 ± 6.17	1.99
CT	Maximize	190.99	245.29	245.51	237.54 ± 9.98	1.40

Means ± standard deviations from three replicates (*n* = 3).

**Table 6:** Antioxidant properties of the optimized extracts.

Assays	Amount
ABTS (mg of TE/g of pericarp)	141.92 ± 10.29
DPPH (mg of TE/g of pericarp)	88.43 ± 1.60
CUPRAC (mg of TE/g of pericarp)	351.59 ± 17.86
RP (mg of TE/g of pericarp)	199.89 ± 4.26
TFC (g of CEs/kg of pericarp)	40.80 ± 1.36

Means ± standard deviations from three replicates (*n* = 3).

**Table 7:** HPLC profile of the optimized extract for various phytochemical compounds (concentration in mg/g of pericarp).

Compound	Amount	Class
Cyanidin-3-glucoside	15.61 ± 0.49 b	Anthocyanin
Delphinidin	16.25 ± 0.28 b	Anthocyanin
Gallic acid	0.70 ± 0.00 d	Phenolic acid
Caffeic acid	14.97 ± 0.18 b	Phenolic acid
Hesperidin	7.42 ± 0.80 c	Phenolic acid
Epicatechin	102.73 ± 1.66 a	Flavonoid
Quercetin	1.38 ± 0.99 d	Flavonoid
Kaempferol	0.37 ± 0.00 d	Flavonoid

Means ± standard deviations from three replicates (*n* = 3). Means followed by the same letter in a column are not different (*p* < 0.05).

**Table 8:** UHPLC-MS analysis for metabolite identification in aqueous and optimized DES extracts.

Name	Class	Structure	RT	*m*/*z* Ratio	Aqueous Extract	DES Optimized Extract
Eriodictyol	Flavonoid	C_15_H_12_O_6_	0.61	289.07067	+	−
Luteolin-7-glucoside	Flavonoid	C_21_H_20_O_11_	0.67	449.10845	+	+
Vanillic acid	Phenolic acid	C_8_H_8_O_4_	1.37	169.04957	+	−
Luteolin-7-glucoside	Flavonoid	C_21_H_20_O_11_	1.55	449.1084	−	+
Prunin	Flavonoid	C_21_H_22_O_10_	1.99	435.12873	+	+
Cyanidin-3-O-galactoside	Anthocyanin	C_21_H_20_O_11_	2.32	449.10873	+	+
6-Hydroxycoumarin	Coumarin	C_9_H_6_O_3_	2.32	163.03933	+	+
Quercetin-4′-O-glucoside	Flavonoid	C_21_H_20_O_12_	2.32	465.10399	+	+
Dihydrorobinetin	Flavonoid	C_15_H_12_O_7_	2.48	305.06551	−	+
Peonidin-3-O-beta-galactoside	Anthocyanin	C_22_H_22_O_11_	2.49	463.12452	−	+
Kaempferol-3-O-xyloside	Flavonoid	C_20_H_18_O_10_	2.57	419.09765	−	+
Kaempferol-3-robinobioside	Flavonoid	C_27_H_30_O_15_	2.62	595.16693	+	+
Pelargonidin-3-O-glucoside	Anthocyanin	C_21_H_20_O_10_	2.83	433.11357	+	+
Pelargonidin	Anthocyanin	C_15_H_11_CLO_5_	2.83	271.06028	+	+
Quercetin-3-(6″-malonyl)-Glucoside	Flavonoid	C_24_H_22_O_15_	3.04	551.10479	+	+
Cyanidin-3-malonylglucoside	Anthocyanin	C_24_H_22_O_14_	3.11	535.10919	+	+
Cyanidin 3,5-diglucoside	Anthocyanin	C_27_H_31_O_16_	3.11	611.15837	−	+
3-Hydroxy-4-methoxycinnamic acid	Phenolic acid	C_10_H_10_O_4_	3.14	177.05453	+	+
Kaempferol-3-O-xyloside	Flavonoid	C_20_H_18_O_10_	3.17	419.09775	+	+
Peonidin-3-O-beta-galactoside	Anthocyanin	C_22_H_22_O_11_	3.22	463.12427	+	+
Peonidin	Anthocyanin	C_16_H_13_O_6_	3.22	301.07089	+	+
3,5-Dimethoxy-4-hydroxycinnamic acid	Phenolic acid	C_11_H_12_O_5_	3.58	207.05638	−	+
6″-O-Malonylisoquercitrin	Flavonoid	C_24_H_22_O_15_	3.59	551.10341	+	+
Malonylgenistin	Iosflavone	C_24_H_22_O_13_	3.81	519.11458	+	+
Malonylgenistin	Iosflavone	C_24_H_22_O_13_	4.19	519.11406	+	+
Peonidin-3-(malonylglucoside)	Anthocyanin	C_25_H_24_O_14_	4.2	549.12487	+	+
Schaftoside	Flavonoid	C_26_H_28_O_14_	4.36	565.15606	+	+
Cyanidin-3-(dimaonylglucoside)	Anthocyanin	C_24_H_22_O_14_	4.41	621.10976	+	+
Flavone base-3O, 1MeO, O-MalonylHex	Flavonoid	C_25_H_24_O_14_	4.54	549.1249	+	+
Quercetin	Flavonoid	C_15_H_10_O_7_	4.58	303.05013	+	+
Taxifolin	Flavonol	C_15_H_12_O_7_	4.64	305.06575	+	−
Naringenin	Flavonoid	C_15_H_12_O_5_	4.86	273.07575	+	+
Delphinidin-3-galactoside	Anthocyanin	C_21_H_20_O_12_	5.02	465.103	+	+
Rutin	Flavonoid	C_27_H_30_O_16_	5.02	611.1615	+	+
Pelargonidin-3-(dimalonylglucoside)	Anthocyanin	C_27_H_25_O_16_	5.11	605.115	+	+
Skimmin	Coumarin	C_15_H_16_O_8_	5.35	325.09218	+	+
Hesperetin	Flavonoid	C_16_H_14_O_6_	5.52	303.0865	+	+
7,2′,3′-Trimethoxyflavone	Flavonoid	C_18_H_16_O_6_	6.01	313.09272	−	+
Diosmetin	Flavonoid	C_16_H_12_O_6_	6.24	301.07135	+	−
Isorhamnetin-3-O-rutinoside	Flavonoid	C_28_H_32_O_16_	6.35	625.17684	+	+
Isorhamnetin	Flavonoid	C_16_H_12_O_7_	6.57	317.06583	+	+
Hesperetin	Flavonoid	C_16_H_14_O_6_	7.03	303.0866	−	+
7-Hydroxy-4-methoxymethyl coumarin	Phenolic acid	C_11_H_10_O_4_	7.49	207.06538	−	+
Feruloyl tyramine	Phenolic acid	C_18_H_19_NO_4_	8.23	314.13905	+	+
Quercetin	Flavonoid	C_15_H_10_O_7_	8.26	303.04999	+	+
Cirsimaritin	Flavonoid	C_17_H_14_O_6_	8.37	315.0873	+	+
Diferuloyl putrescine	Phenolic acid	C_24_H_28_N_2_O_6_	8.81	441.20299	+	+
Pulicarin	Flavonoid	C_20_H_16_O_8_	9.15	375.10823	+	+
Feruloyl putrescine	Phenolic acid	C_14_H_20_N_2_O_3_	9.2	265.15437	+	+
Diferuloyl putrescine	Phenolic acid	C_24_H_28_N_2_O_6_	9.27	441.20273	+	+
Homoeriodictyol	Flavonoid	C_16_H_14_O_6_	10.18	303.08666	+	+
Chryseriol	Flavonoid	C_16_H_12_O_6_	10.63	301.07188	+	+
Tricin	Flavonoid	C_17_H_14_O_7_	10.76	331.08175	+	+
Isorhamnetin	Flavonoid	C_16_H_12_O_7_	10.79	317.06613	+	+
Pinolenic acid	Phenolic acid	C_18_H_30_O_2_	22.65	279.2325	+	−
Erucamide	Fatty acid	C_22_H_43_NO	33.19	338.34218	+	+
Ferulic acid	Phenolic acid	C_10_H_10_O_4_	34.41	195.06512	+	+

Note: “RT” stands for retention time; “+” indicates the presence of the compound; “−” indicates the absence of the compound.

## Data Availability

Data are contained within the article and Supplementary Materials.

## Supplementary Materials

The following supporting information can be downloaded at https://www.mdpi.com/article/10.3390/antiox14010009/s1, Figure S1. (A–F) Response surface plots for interactions between time, temperature, water, amplitude and solid-to-liquid ratio on TPC extraction using DES and UAE; Figure S2. (A–F) Response surface plots for interactions between time, temperature, water, amplitude and solid-to-liquid ratio on CT extraction using DES and UAE; and Figure S3. (A) Comparison between the predicted and experimental values for total anthocyanin content and (B) Perturbation plot for total anthocyanin content..
