# Supplementary material for: Optimization of Polyphenol Extraction from Purple Corn Pericarp Using Glycerol/Lactic Acid-Based Deep Eutectic Solvent in Combination with Ultrasound-Assisted Extraction
Source: Antioxidants (Basel). 2024 Dec 25;14(1):9. doi: 10.3390/antiox14010009 (PMC11762350; doi:10.3390/antiox14010009)

Supplementary Figure. S1. (A-F) Response surface plots for interactions between time, temperature, water, amplitude and solid-to-liquid ratio on TPC extraction using DES and UAE.

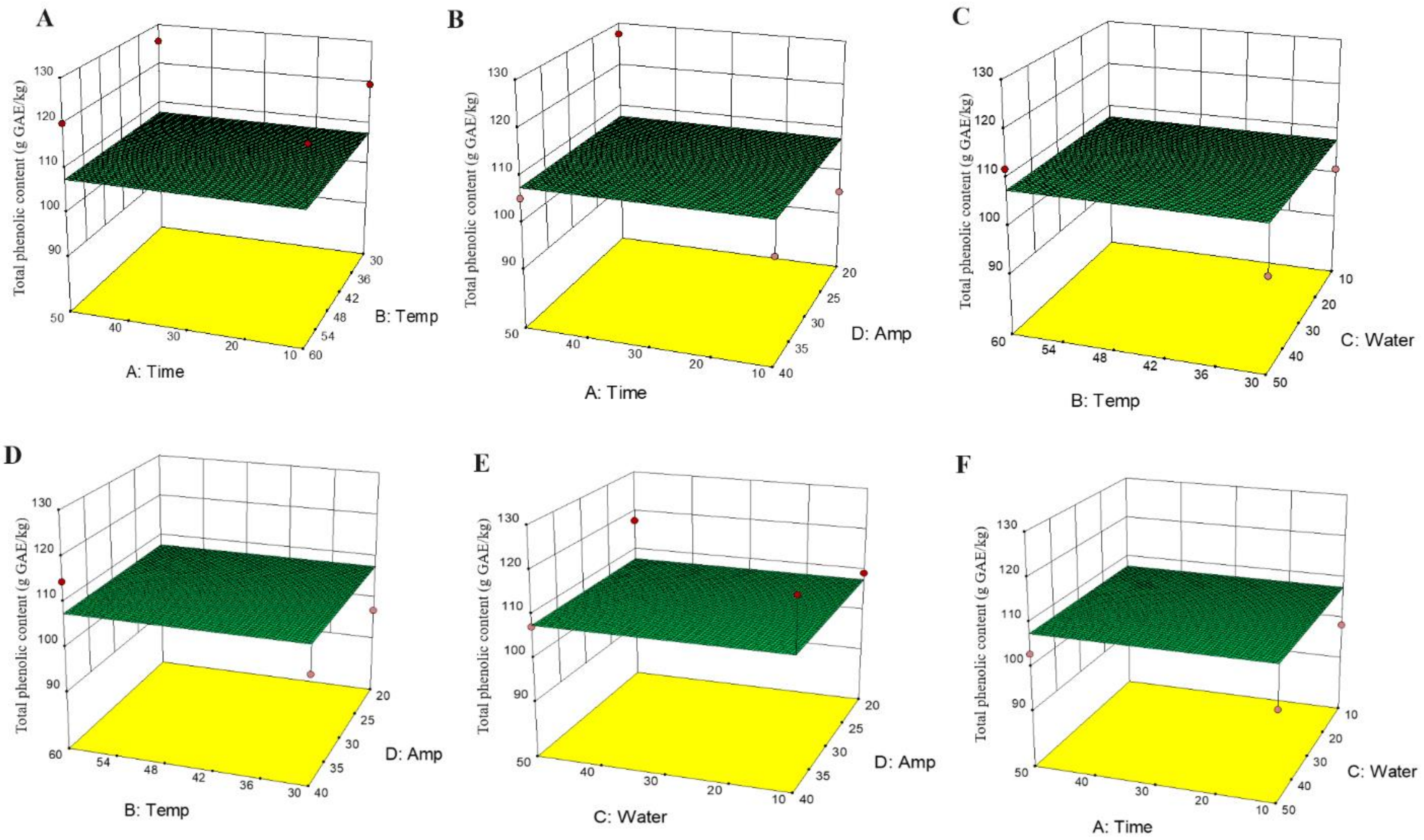

Supplementary Figure. S2. (A-F) Response surface plots for interactions between time, temperature, water, amplitude and solid-to-liquid ratio on CT extraction using DES and UAE.

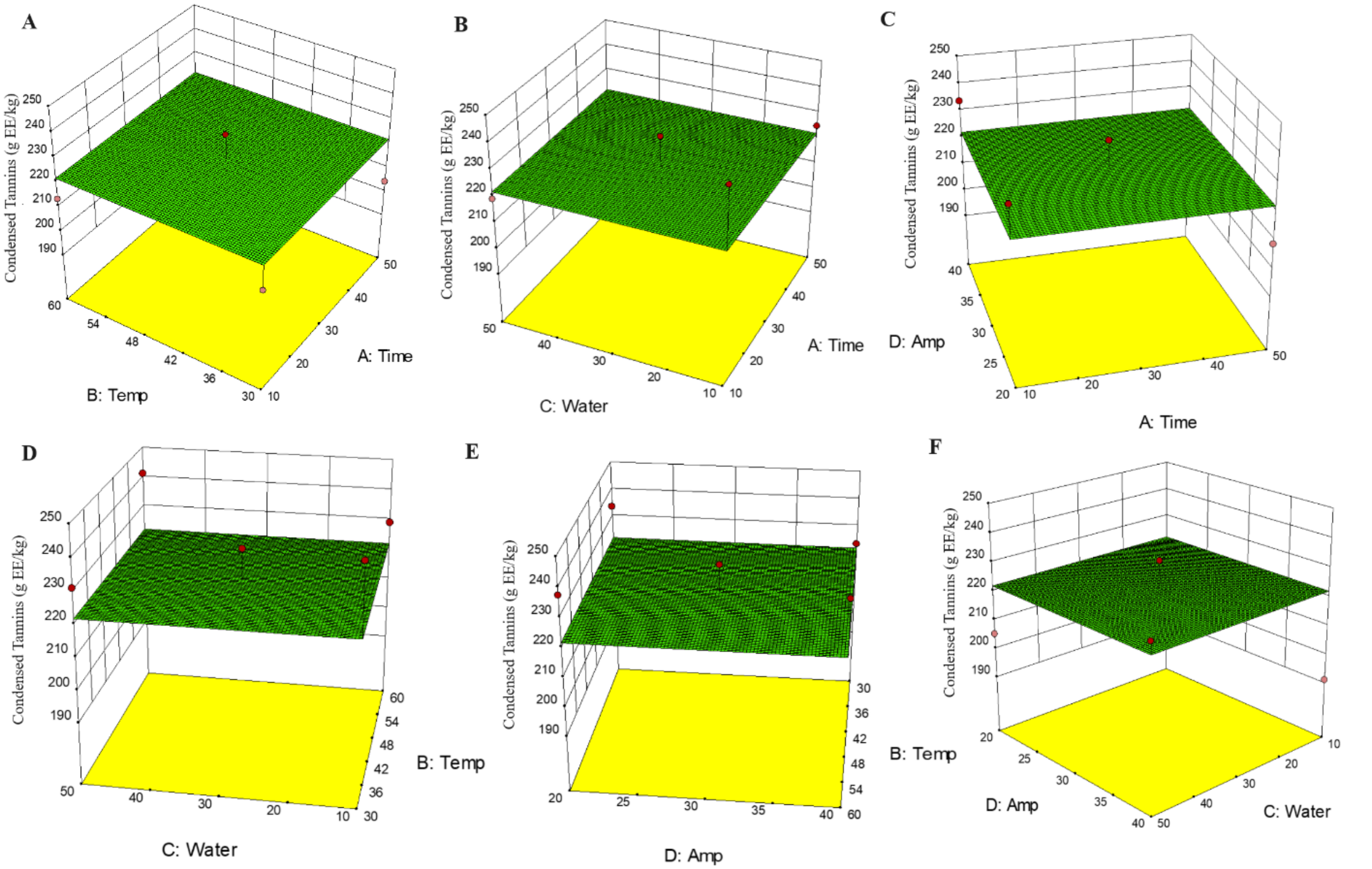

Supplementary Figure. S3. (A) Comparison between the predicted and experimental values for total anthocyanin content and (B) Perturbation plot for total anthocyanin content.

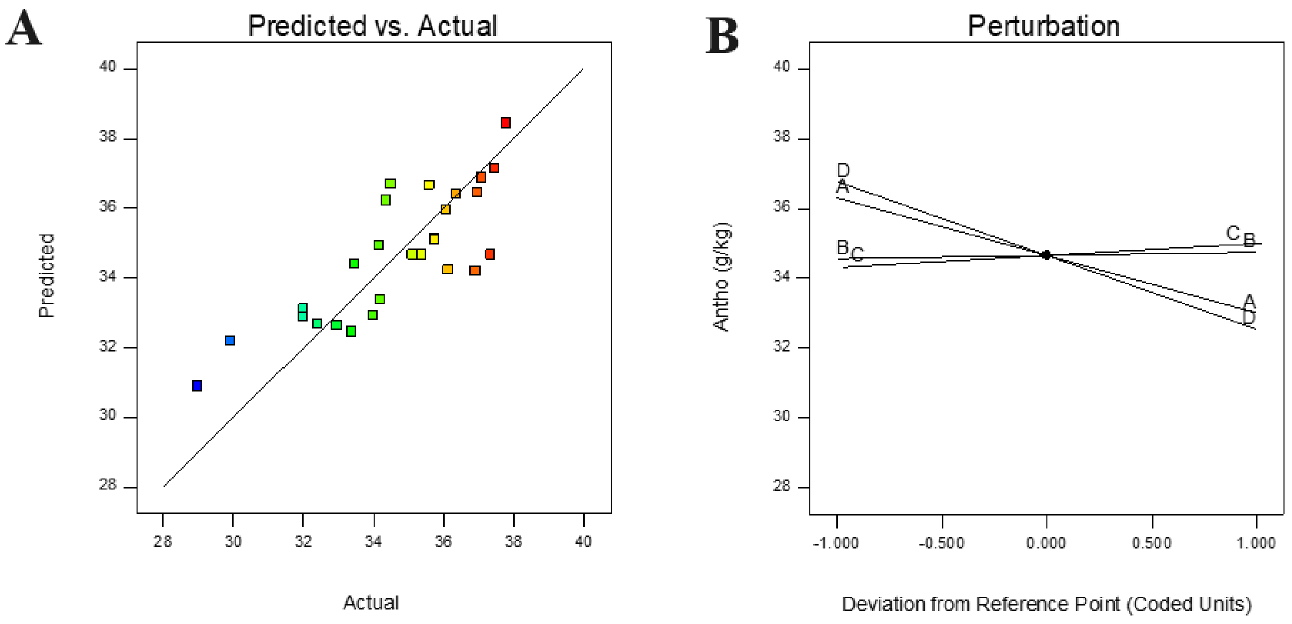

Supplement: Supplementary file 1 [file antioxidants-14-00009-s001.zip › antioxidants-3370642-supplementary.pdf]
